# Oral pigmented lesions: a retrospective analysis from Brazil

**DOI:** 10.4317/medoral.24168

**Published:** 2020-08-27

**Authors:** Danielle Mendes da Silva Albuquerque, John Lennon Silva Cunha, Ana Luiza Oliveira Corrêa Roza, Lady Paola Aristizabal Arboleda, Alan Roger Santos-Silva, Marcio Ajudarte Lopes, Pablo Agustin Vargas, Jacks Jorge, Oslei Paes de Almeida, Aline Corrêa Abrahão, Michelle Agostini, Mário José Romañach, Bruno Augusto Benevenuto de Andrade

**Affiliations:** 1 DDS, MSc. Department of Oral Diagnosis and Pathology, School of Dentistry, Federal University of Rio de Janeiro (UFRJ), Brazil; 2 DDS, MSc student. Department of Oral Diagnosis, Piracicaba Dental School, University of Campinas (UNICAMP), SP, Brazil; 3 DDS, PhD student. Department of Oral Diagnosis, Piracicaba Dental School, University of Campinas (UNICAMP), SP, Brazil; 4 DDS, PhD. Department of Oral Diagnosis, Piracicaba Dental School, University of Campinas (UNICAMP), SP, Brazil; 5 DDS, PhD. Department of Oral Diagnosis and Pathology, School of Dentistry, Federal University of Rio de Janeiro (UFRJ), Brazil

## Abstract

**Background:**

Pigmented lesions are uncommon in the oral mucosa, and studies investigating the incidence and types of these lesions are desired to improve the diagnostic knowledge of clinicians. The aim of this study was to analyze the distribution of oral pigmented lesions in a Brazilian population.

**Material and Methods:**

A retrospective descriptive cross-sectional study was performed. Oral pigmented lesions were retrieved from the files of two oral and maxillofacial pathology services from Brazil over a 45-year period (1974-2019). The clinical data and the diagnoses of each case were retrieved and included in a Microsoft Excel® database.

**Results:**

From 77.074 lesions diagnosed in this period, 761 (0.99%) represented pigmented lesions of the oral mucosa, including 351 (46.1%) melanocytic and 410 (53.9%) non-melanocytic lesions, with a higher incidence in females (73.2%) between the fourth and seventh decades of life. Amalgam tattoo (53.6%) represented the most common lesion, followed by melanotic macule (18.3%) and racial pigmentation (10.8%). Other pigmented lesions included nevus (9.9%), post-inflammatory pigmentation (3%), melanoma (2.1%), melanoacanthoma (1.4%), smoker's melanosis (0.4%), drug-induced pigmentation (0.3%), and melanotic neuroectodermal tumor of infancy (0.1%). The buccal mucosa was the most commonly affected site (25.2%), followed by the alveolar ridge (14.5%), and gingiva (11.8%).

**Conclusions:**

The current findings were similar to previous studies with minor differences due methodology and characteristics of the services from where lesions were retrieved. The knowledge of these data may contribute to a better understanding of oral pigmented lesions and assist clinicians to better recognize and manage them.

** Key words:**Pigmented lesions, pigmentation, melanin, amalgam, oral cavity.

## Introduction

Pigmented lesions of the oral cavity are uncommon and might have a melanocytic or a nonmelanocytic origin (1,2). They can be classified clinically into focal pigmentations such as oral melanotic macule, amalgam tattoo, melanocytic nevus, melanoacanthoma, melanotic neuroectodermal tumor of infancy, melanoma, and multifocal or diffused pigmentations, including entities such as physiologic (racial) pigmentation, drug-induced hyperpigmentation, smoking-associated melanosis, post-inflammatory pigmentation, heavy metal pigmentation, and melanosis associated with systemic diseases such as Addison disease, neurofibromatosis, Peutz-Jeghers syndrome, McCune-Albright syndrome, Carney complex syndrome, and Bannayan-Ruvalcaba-Riley syndrome (3-14).

Usually, oral pigmented lesions might be clinically diagnosed and monitored, or even biopsied, since the diagnosis cannot always be established based only on clinical examination, particularly because of their varied clinical appearance (3,4). According to the few large sample studies of oral pigmented lesions published in the English-language literature, their prevalence provided from biopsies comprises less than 2% of all oral diagnoses (1). The knowledge of oral pigmented lesions through epidemiologic studies can help to better understand their prevalence, incidence and evolution and determine eventual regional and global differences (3-5).

The objective of this study is to report an additional large series and analyze the distribution of oral pigmented lesions retrieved from two reference centers on oral and maxillofacial pathology of southeastern region of Brazil.

## Material and Methods

In the present study, a retrospective analysis of oral pigmented lesions was conducted from the archives of two Brazilian oral and maxillofacial pathology services: Department of Oral Diagnosis, School of Dentistry of Piracicaba, University of Campinas (UNICAMP); and Department of Oral Diagnosis and Pathology, School of Dentistry of the Federal University of Rio de Janeiro (UFRJ) over a 45-year period (from January 1974 to December 2019). Diagnoses, age, gender, and anatomical location of each case were retrieved from the services files and tabulated in Microsoft Excel®. All cutaneous cases, including lip lesions involving the skin, were excluded from the study. The search of oral pigmented lesions included amalgam tattoo, oral melanotic macule, racial pigmentation, junctional nevus, compound nevus, intramucosal nevus, blue nevus, nevus (not specified), linear verrucous epidermal nevus, post-inflammatory pigmentation, melanoma, melanoacanthoma, smoker's melanosis, drug-induced pigmentation, and melanotic neuroectodermal tumor of infancy.

Descriptive and quantitative data analysis was performed using the Statistical Package for the Social Sciences for Windows 20.0 (SPSS, Inc., Chicago, IL, USA). Continuous variables were expressed as mean, median, and standard deviation values. Categorical variables were expressed as the absolute number of cases and percentage values. Fisher’s exact test was used to evaluating the association between clinical and demographic characteristics, adopting a *p-value* of ≤0.05 and 95% confidence interval.

## Results

**Figure 1:**
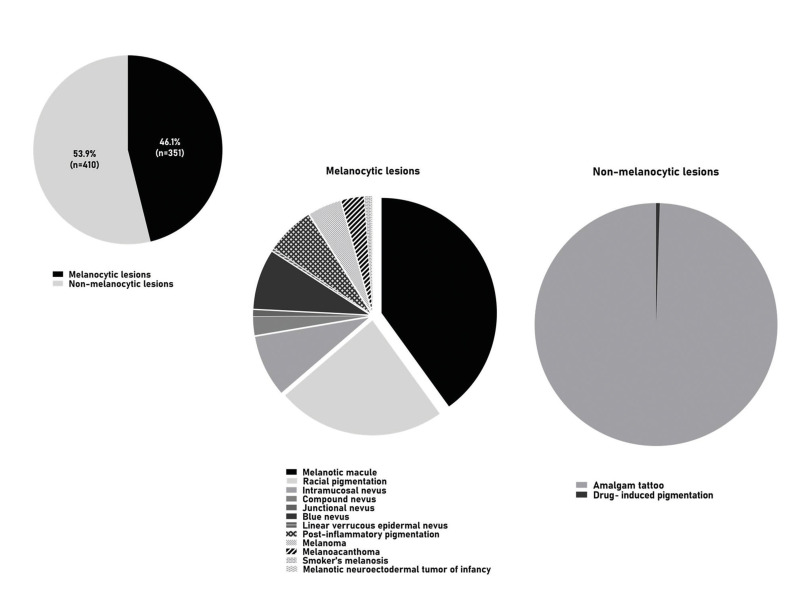
Oral pigmented lesions detected in the study.

**Figure 2:**
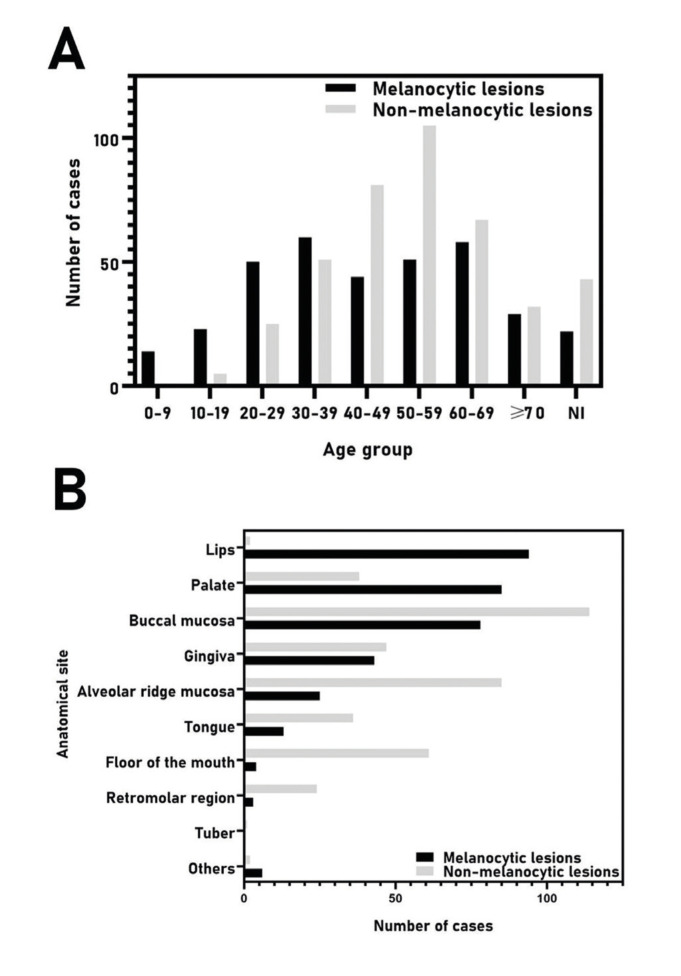
A. Distribution of 761 oral pigmented lesions according to the age group (decade of life) and B. primary site of involvement. NI, not informed.

**Figure 3:**
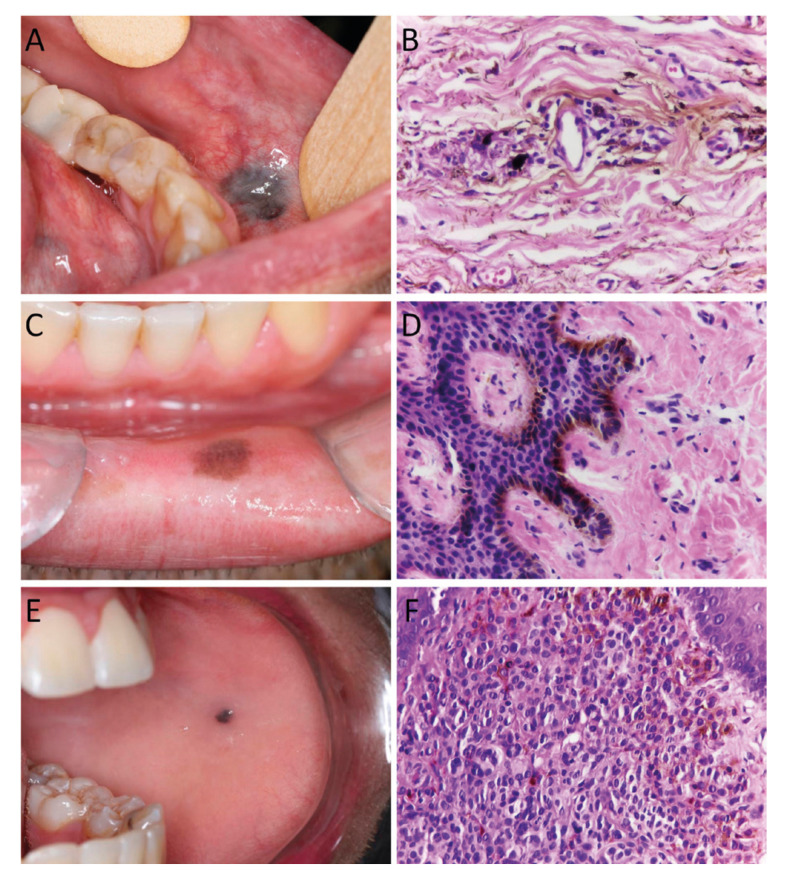
Clinical and histopathological features of oral pigmented lesions. A. Amalgam tattoo located in the transition between buccal mucosa and lower lip. B. Presence of fine black and brown amalgam particles along collagen bundles and around blood vessel (Hematoxylin and eosin, 400X). C. Melanotic macule in the lower lip presenting as a small and well-circumscribed, brown macule. D. Increased production of melanin by melanocytes located in the basal layer (Hematoxylin and eosin, 400X). E. Oral melanocytic nevus located in the left buccal mucosa. F. Presence of pigmented nevus cells in the lamina propria (Hematoxylin and eosin, 400X).

**Figure 4:**
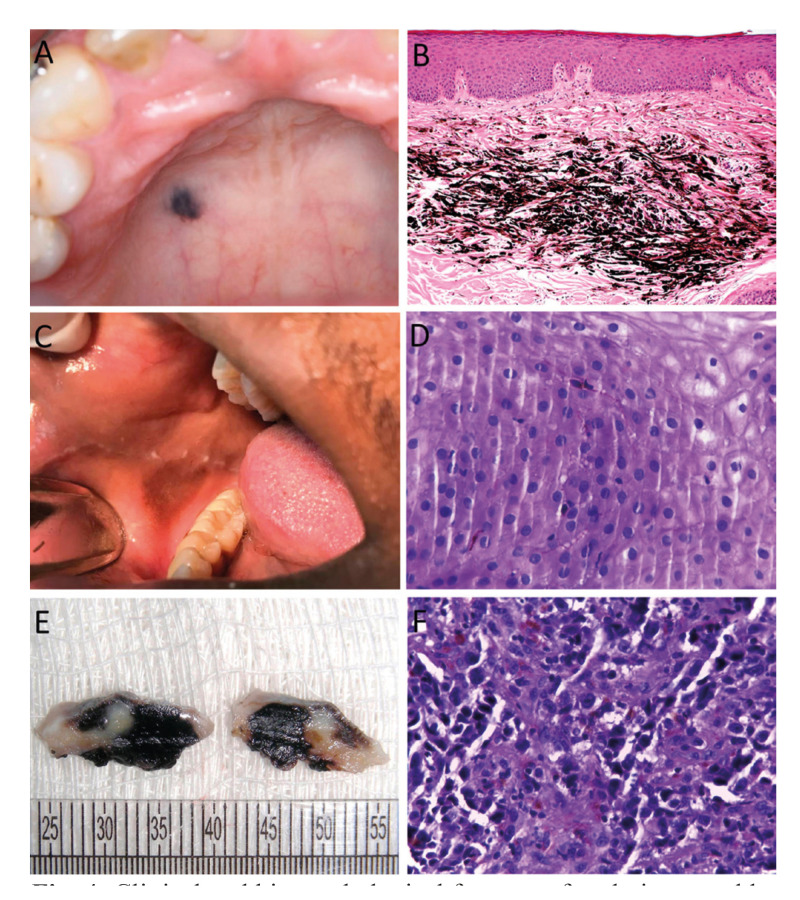
Clinical and histopathological features of oral pigmented lesions. A. Blue nevus in the hard palate. B. Presence of spindle melanocytes in deep portion of the connective tissue (Hematoxylin and eosin, 200X). C. Melanoacanthoma in the right buccal mucosa. D. Presence of pigmented dendritic melanocytes throughout the epithelium (Hematoxylin and eosin, 400X). E. Gross appearance of an incisional biopsy of oral melanoma with darkish cut surface and fibroelastic consistency. F. Proliferation of malignant melanocytes with melanin production (Hematoxylin and eosin, 400X).

The two oral and maxillofacial pathology services received 77.074 surgical specimens between 1974-2019, of which 761 represented pigmented lesions of the oral mucosa (0.99%), including 351 (46.1%) melanocytic and 410 (53.9%) non-melanocytic lesions, distributed among fifteen diagnosis (Fig. 1, Fig. 2, Fig. 3 and Fig. 4).

The majority of patients were female (n=557, 73.2%) with an overall female:male ratio of 2.7:1 (Table 1). Most lesions occurred in patients between the fourth and seventh decades of life (Fig. 2), with a mean age of 47.6±17.5 years (range 0-93 years). Table 2 shows the distribution of each pigmented lesion, according to the age of patients. Regarding the anatomical site, the buccal mucosa was the most commonly affected, with a frequency of 25.2% (n=192), followed by the alveolar ridge (n=110, 14.5%), and gingiva (n=90, 11.8%) (Fig. 2). Both melanocytic and non-melanocytic lesions predominated in the buccal mucosa (22.2% and 27.8%, respectively) (Table 3).

Among the melanocytic lesions (n=351), melanotic macule was the most frequent (n=139; 39.6%), followed by racial pigmentation (n=82, 23.4%), and intramucosal nevus (n=30, 8.5%) (Table 1). Considering only the intraoral nevi (n=76), the following morphological subtypes were found: intramucosal (n=30, 39.5%), blue nevus (n=29, 38.2%), compound (n=9, 11.8%), junctional (n=3, 3.9%), and nevus (not specified; n=4, 5.3%). In addittion, a rare case of linear verrucous epidermal nevus with involvement of the oral mucosa was found. The melanocytic lesions were diagnosed mainly between the fourth and fifth decades of life (Fig. 2); however, the age ranged from 0 to 87 years, with an average age of 44.0 years (SD±19.6) (Table 2). Most cases occurred in the buccal mucosa (n=78, 22.2%), followed by lower lip (n=74, 21.1%), and hard palate (n=63, 17.9%) (Table 3) of female patients (n=245; 69.8%), with a female:male ratio of 2.3:1.

**Table 1:**
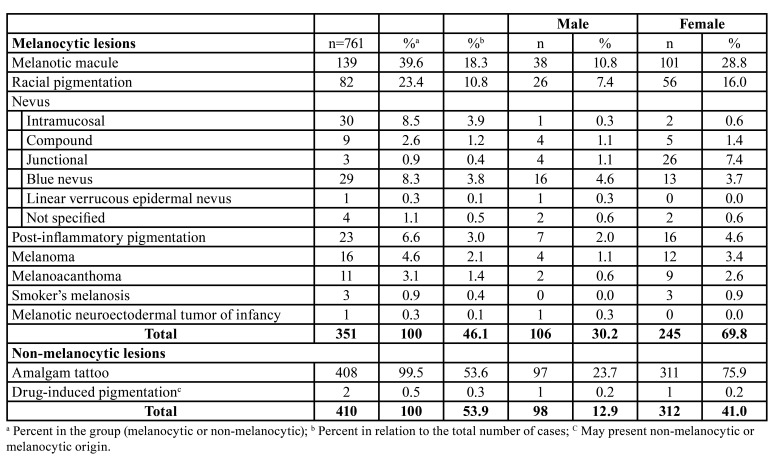
Diagnosis and gender distribution of 761 oral pigmented lesions.

**Table 2:**
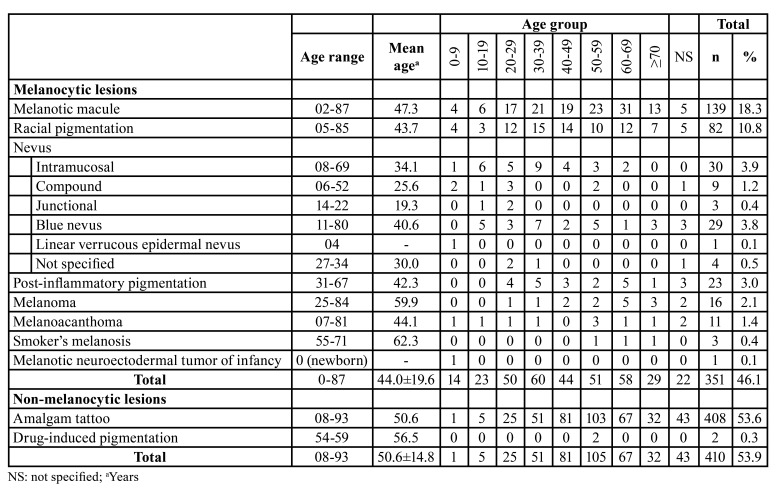
Age group distribution (decade of life) of 761 oral pigmented lesions.

**Table 3:**
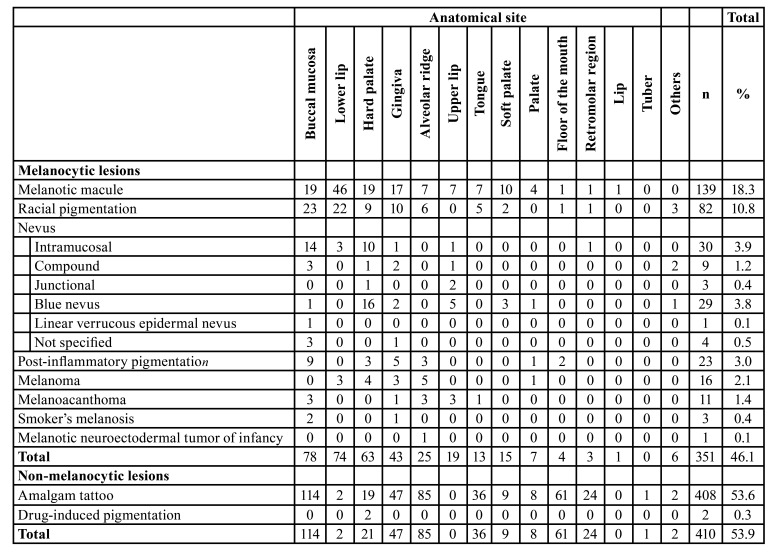
Distribution of the 761 oral pigmented lesions according to the location.

Regarding the non-melanocytic lesions (n=410), amalgam tattoo was the most frequent (n=408, 99.5%), followed by drug-induced pigmentation associated with chronic chloroquine therapy, but with only 2 cases (Table 1). The age ranged from 8 to 93 years, with a mean age of 50.6 years (SD±14.8) (Table 2). Most cases also occurred in the buccal mucosa (n=114, 27.8%) and alveolar ridge mucosa (n=85, 20.7%) in female patients (n=312; 76.1%), with a female:male ratio of 2.7:1 (Table 2 and Table 3).

When melanocytic vs. non-melanocytic lesions were compared, the buccal mucosa was the most affected site mainly by non-melanocytic lesions (*p*<0.0001). Also, the non-melanocytic lesions were more common in adult and older patients (*p*<0.0001); results were statistically significant (Table 4).

**Table 4:**
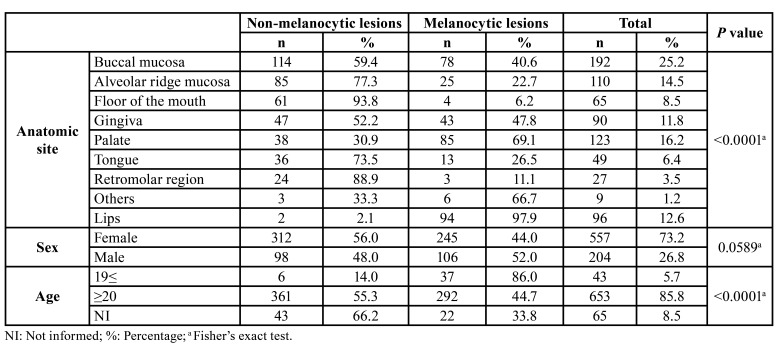
Anatomic site, gender, and age group distribution of 761 melanocytic and non-melanocytic lesions.

## Discussion

To the best of our knowledge, this study provides the largest oral pigmented lesions series in a population from Brazil, and, the second largest in the English-language literature (1,3-5). Buchner *et al*. (4) had a larger sample of 773 cases, but only focusing solitary melanocytic oral lesions. Pigmented lesions affecting oral mucosa are relatively uncommon in oral pathology specialized diagnostic centers representing about 1-2% of all histopathological specimens, also demonstrating a wide variability of entities recognized (1,3-5). In the current series oral pigmented lesion corresponded to 0.99% of all cases.

Amalgam tattoo represented the most common oral pigmented lesion in the present study, followed by melanotic macule and racial pigmentation. Similar results were also observed by Tavares *et al*. (1) which also found amalgam tattoo to be the most common entity (46.3%), followed by melanotic macule (22.9%), and oral nevi (20.5%). It must be considered that oral mucosa racial pigmentation and smoker’s melanosis are diagnosed clinically and not biopsied. Therefore there is an underestimation of the incidence of these lesions when the study is based of pathological specimens as performed in this report. For example, Hassona *et al*. (3) reported a clinical study evaluating 1.275 patients, and found that 30.3% showed some oral pigmentation, mainly represented by racial pigmentation (39.9%) and smoker’s melanosis (32.9%), while amalgam tattoo (18.9%), melanotic macule (5.7%), post-inflammatory pigmentation (1.6%), medications or systemic diseases (0.52%), heavy metal deposition (0.26%), and nevus (0.26%) were also detected but in lower prevalence.

Interestingly, most lesions with exception of blue nevus, linear verrucous epidermal nevus and melanotic neuroectodermal tumor of infancy were more common in women. The higher prevalence of oral pigmented lesion in female in our series (73.2%) has also been previously reported (1,3,4,14). The female predominance may be consequence of the awareness with their oral health, since a higher frequency of females is found in adult populations, possibly representing the higher concern demonstrated by females than males (3,4).

Amalgam tattoo is a relatively common finding in the oral mucosa that represents introduction of dental amalgam particles into soft tissue during restorative or surgical procedures, and usually manifests as an isolated bluish or black macule in various areas of the mucosa (11,13). In the present study, amalgam tattoo was mainly found in females between the fourth and sixth decade of life, with lesions mainly in the buccal mucosa and alveolar ridge, which are in accordance with previous reports that showed about 65% of all patients were females, with a mean age of 43-47 years (13). As amalgam tattoo may clinically resemble oral nevi or eventually melanoma, mainly when there is not a radiographic evidence of the metal particles, microscopic examination is usually required, which can explain the high prevalence of the lesion in the present study. Interestingly, the declining frequency of amalgam tattoo in recent years certainly is associated with the use of esthetical restorative materials.

Oral melanotic macule is a small, well-circumscribed, brown-to-black pigmentation that occurs commonly on the lips and gingiva, followed by the palate and buccal mucosa in a wide range of age, showing predilection for females (2-4). In the present study, 139 cases of melanotic macules were identified, mainly on the lower lip mucosa of females in the fifth decade of life. The study of Buchner *et al*. (4) reported the largest sample of oral melanotic macule in the literature, with 665 cases, mainly found at lower vermilion border, gingiva, and palate. Patients’ ages vary considerably, and the fifth decade was the most affected, and the female-to-male ratio was 1.9:1 (4). These features are in accordance with the present study.

Oral melanocytic nevi is a benign neoplasm composed of cells derived from the neural crest, called nevus cells, being less common in the oral mucosa than on the skin, and histologically classified into junctional, compound, intramucosal and common blue nevus (12,14). In the present study intramucosal nevi were the most common type, followed by blue nevi and compound nevi. Junctional nevi were relatively uncommon. Interestingly a case of linear verrucous epidermal nevus was observed. The most common site was the hard palate, followed by the buccal mucosa mainly in females in the fourth decade of live. According to previous studies, the intramucosal is the most common type of oral nevi, accounting for approximately 60% of all cases, and are most commonly found in the palate and buccal mucosa, with patients diagnosed at their third and fourth decades of life (1,12,14).

Inflammatory diseases, such as oral lichen planus, pemphigus, pemphigoid or chronic periodontal disease can cause mucosal pigmentation, so called post-inflammatory pigmentation that can be seen more frequently in dark-skinned individuals (2,10). Clinically, multiple brown to black pigmented areas are noted adjacent to reticular, erosive or vesicular lesions, characterized by increased production of melanin by the melanocytes and accumulation of melanophages in the subepithelial connective tissue (2). Generally, the resolution of the inflammatory process allows the improvement of oral pigmentation (2). In the present study it was observed 23 cases occurring mainly in females in their fourth and seventh decades of life, like observed in previous studies (2,10).

Oral melanoacanthoma is a rare acquired pigmented lesion characterized by dendritic melanocytes dispersed throughout an acanthotic epithelium, being the buccal mucosa and gingiva the most common location (9). It is considered to be a reactive process unrelated to the neoplastic melanoacanthoma of the skin (2). The oral lesions generally regress after removal of traumatic irritants or after biopsy (9). About 45 cases of oral melanoacanthoma have been reported in the literature and according to Buchner *et al*. (4) and Carlos-Bregni *et al*. (9) lesions mainly affected the buccal mucosa of female patients in their fourth decade of life as also observed in our cases. To the best of our knowledge our study showed the highest number of oral melanoacanthoma among all oral pigmented series presented in the English-language literature (1,4,9).

Although the majority of oral pigmented lesions are benign and require minimal intervention, it should be recognized that oral malignant tumors do occur. Oral melanoma is a rare lesion formed by malignant melanocytes, comprising 0.4-1.8% of all melanomas and 0.5% of oral malignancies, being more common in the palate and gingiva of female adult patients (8). Most oral melanomas are heavily pigmented clinically and microscopically, but some are amelanotic and immunohistochemistry for S-100 protein, HMB-45 and Melan-A can be helpful to confirm the diagnosis of these cases (8). In the present study, 16 cases of oral melanoma were identified with demographic and clinical data similar to previous studies (1,8).

In summary, this is the largest series of oral pigmented lesions reported in Brazil, and amalgam tattoo, melanotic macule and racial pigmentation were the most common oral lesions, occurring mainly in women between the fourth and seventh decades and affecting mainly particularly the buccal mucosa and the alveolar ridge. Since retrospective studies of large series of oral pigmented lesions are scarce in the literature, these data may contribute to the better understanding by oral pathologists, clinicians and different medical specialists who first deal with these patients.
